# Environmentally driven migration in EU discourse: norms, policies and realities

**DOI:** 10.14324/111.444/ucloe.1975

**Published:** 2024-03-26

**Authors:** Lucia Wirthová

**Affiliations:** 1 Comenius University, Faculty of Social and Economic Sciences, Institute of European Studies and International Relations, Bratislava, Slovakia

**Keywords:** European Union, migration, climate change, normative power, securitisation, discourse analysis

## Abstract

For decades, the European Union (EU) has been addressing issues related to climate change and ecological degradation as a self-proclaimed pro-environmental and human rights-oriented actor. Correspondingly, the topic of the so-called *environmentally driven migration* entered the EU discourse at the dawn of the new millennium. Considering the EU’s interest in the human rights and environmental/climate issue areas, I argue it is important to ask what the Union’s approach to this matter has been. Thus, this article assesses the European Union discourse related to the topic of environmental migration over the past 20-year period. Through the theoretical lens of the Copenhagen School of Security Studies and the normative power EU conception, this paper critically analyses the EU’s securitisation of climate change in relation to persons referred to as environmental migrants. Based on a qualitative discourse analysis, the preliminary results imply that the topic has been receding into the background of the EU agenda. In line, environmental migrants have been pushed aside by a multiplicity of other subjects threatened by climate change, thus receiving lesser attention in the EU climate change and migration management policies. Overall, the findings show a shift from an alarmist discourse to more pragmatism on the EU’s behalf and a larger focus on adaptation and resilience in most affected areas. With this in mind, this article questions the normative standard the EU sets for itself when it comes to the case of environmental migrant protection.

## Introduction

As a self-proclaimed leader against threats and consequences posed by climate change and environmental degradation, the European Union (EU) has been attempting to raise awareness and gain support in the fight against such threats within as well as beyond EU borders [1–4]. Equally, the EU continuously aims to be an ardent international human rights protector [5,6]. Hence, the main goal of this article is to critically examine the self-assumed EU normativeness on a case which combines the two issue areas [7]. By applying Manners’ normative power EU (NPE) concept and the securitisation approach of the Copenhagen School of Security Studies, I assess the EU approach to what tends to be referred as environmental migration between the years 1999 and 2019. In turn, a qualitative discourse analysis (QDA) applied to 20 years of EU discourse provides insight into the (non-)reflection of the assumed environmental migration in the EU agenda for climate change and migration management. This article therefore addresses the question of whether the EU meets the standards it sets for itself on the case of environmental migration. By doing so it contributes to the existing literature on the topic of EU power conceptions including NPE [8–12], on (de-)securitisation [13–17] and (environmental) migration [18–21]. Likewise, it provides insight on the application of qualitative discourse analysis methods in migration discourses [22–26]. The article starts with a concise explanation of the so-called environmental migration phenomenon. From there it goes on to the overview of the three main conceptions of European power in foreign policy. In particular, it highlights the connection between NPE and securitisation, as defined by the Copenhagen School of Security Studies. A methodological section follows, describing the QDA application. The last part of the article explains the results and elaborates the relevance of the drawn conclusions in the post-analysis period.

In sum, exploring the impact of environmental factors, particularly climate change, on human migration during the 20th and 21st centuries is the focal point of this paper. While it remains difficult to infer and quantify direct causal relationships between environmental/climate drivers and human movements, due to the advancing environmental stresses it nevertheless remains a topic of discussion in international affairs. The article therefore highlights the importance of considering the evolving environmental dynamics in migration policies and approaches.

## Environmental migration as a phenomenon?

As such, environmental migration was internationally recognised as a phenomenon since the 1970s, but it entered the EU discourse only in the year 1999 [27,28]. When the EU took interest in the topic and started analysing it on its own throughout the early 2000s [29], the international context surrounding environmental migrants had become alarmist already. It was also rather pessimistic about their future [21]. The most referenced reports at the time, for example, the UK Stern Review (2006) or *World in Transition – Climate Change as a Security Risk* by the German Advisory Council (2007), foresaw hundreds of millions of environmental migrants by the year 2050. Back then, quantitative analyses commonly predicted around 200–250 million environmental migrants [30–33], while others expected as many as one billion [34]. Accordingly, politicians and certain scholars warned of an *approaching crisis* [35–37]. Some such as Myers (1995) called it a *global environmental exodus* as cited in Baldwin, Methmann and Rothe [30], while the Deputy High Commissioner for the United Nations High Commissioner for Refugees (2008) publicly spoke of an ensuing *global-scale emergency* [38]. The Council of Europe (2008) warned the international community not to ignore the issue, which could become one of the ‘greatest demographic and humanitarian challenges of the century worldwide’ [32]. Although the biggest concerns were connected to the least developed countries, called hot spots, Europe was also alerted to the consequences of climate change including environmentally induced migration. Apparently, Europe would be susceptible to these *dangers* too [31,32].

Nonetheless, more recently, the initial alarmist and deterministic narratives have been contested. A growing body of literature has been pointing out the ontological and epistemological fallacy of the concept of environmental migration and the associated ‘solutions to the problem’ [39–42]. Among the most pressing critiques is the fact that identifying persons who move *only* due to environmental (or climate related) reasons is an unattainable task for a number of reasons, namely the issue of causality, whereby the interconnectedness of environmental and socio-economic factors makes it impossible to single out persons affected directly and only by the environment or by climate change [39–46]. This is a very different understanding compared to initial findings, which assumed direct links between environmental changes with negative consequences on living conditions, further enhanced by climate change, and large population movements [47]. In this previous line of thought the most threatened people susceptible to such effects were inhabitants of small island states [48], coastal and deltaic regions, low-lying islands and sub-Saharan Africa [49]. From Homer-Dixon’s 1991 perspective, for example, poor countries would be the ones most impacted by environmental changes and experience population displacement [50].

At the present time, however, the discussions evolved to question why there is lack of a broadly accepted classification, and why labels such as refugees, migrants or displaced persons are misleading [39–42]. Arguably, the relevant actor’s preference and type of discourse may be at the heart of this problem, overestimating the value of Western knowledge systems over local ones [39], unsound justifications for development interventions [51] or reinforcement of populist/negative postcolonial images of the ‘other’ [21,52], often linked to expectations that the ‘Global South’ will migrate to the ‘Global North’ in overwhelming numbers [53]. Another complication is the interchangeable use of the adjectives ‘climate’ and ‘environmental’ for these persons. All of these issues, and many more, contribute to the fact that there is still no universally agreed legal definition [20,45,54–56]. This means that the narratives (mainly in the ‘Global North’) speak of the so-called environmental migrants on the one hand yet fail to clearly conceptualise them on the other hand, all the while pointing out a legal vacuum in which the migrants find themselves. In addition, states, especially European ones, oppose the idea of broadened protection statuses for such migrants [56,57]. This conundrum is a continuing one.

While in this article I refer to *the human beings* as environmental migrants, I do so only because that is one of the terms used in the analysed discourse. It is beyond the scope of this article to delve into the abovementioned intrinsic conceptual assessments. Instead, this article provides an insight into the internal discourse held within the EU about ‘environmental migrants’, where they are viewed as the referents experiencing environmental and climate changes, without further elaboration on the question whether these changes are or are not the sole reasons for the migrants’ movement. By doing so, I aim to highlight the contrast between the self-imposed normative image created by the EU in both environmental and human rights questions versus the language used to describe such persons.

## The three European power conceptions

The argumentation and meaning of this article are built on the normative conception of power and how it is used by the EU in its own discourse about environmental migration. Before the NPE concept was born, however, there were alternative approaches that led to its genesis. Due to its so-called sui generis nature, the EU has always been an outlier when it came to its involvement in foreign policy and the types of power it exerted [58]. Introduced by Duchêne in 1972, the initial conception labelled the EU a civilian power. At the core of this type of power, Duchêne argued, is a strong economic potency and the desire for peaceful resolution of conflicts. In the pursuit of such goals, diplomacy heavily depends on multilateralism and involves international organisations [58]. Another significant feature as to why the concept was created was the fact that the EU did not possess any form of supranational army. Instead, individual states retained control over their military capabilities, which at the same time were comparatively weaker than the American or Soviet ones. According to Maull, who was another proponent of the civilian power concept, post-war Japan and Germany had to accept cooperation with others through peaceful means as it was the best option to achieve their objectives [59]. Equally, the European community as a whole was in a comparable situation and had to rely on a ‘softer’ approach. In turn, trade and development policies came to the forefront of EU foreign policies.

Despite the initial success, in the changing Cold War environment of the 1980s–1990s, the civilian conception became largely criticised by authors such as Bull [60]. The return to power politics underlined the need for hard power, meaning a civilian actor would not stand a chance in the arena against such opponents. Consequently, if the EU wanted to successfully assert its dominance, it had to become stronger in a traditional sense. Reliance on the North Atlantic Treaty Organisation, hence the USA, as the main security provider was seen as insufficient [60]. Moreover, according to the critics, civilian capabilities such as peacekeeping forces were blending with the traditional military capabilities anyway. Labelling the EU as a civilian power was thus seemingly incorrect.

In the early 2000s a third (normative) conception was created in response to both the civilian and military power concepts. As Manners argued in 2002, what makes the EU different from other international actors is its dedication to norms. His suggestion that the EU tries to define standards of ‘good’ behaviour, as well as to define what is ‘normal’ [8]. These goals are based on universal norms and principles derived from the United Nations (UN), namely peace, liberty, democracy, the rule of law and human rights. Furthermore, social solidarity, anti-discrimination, good governance and sustainable development arguably form the rest of the main EU agenda [9]. In terms of foreign policy, the proponents claim, the EU tries to spread these norms abroad through various means. Most importantly, however, it is trying to lead by the normative example, often at the expense of its own benefits [61]. Such a willingness to bind oneself, not only others, to rules and moral practices thus distinguishes EU as a different kind of actor. While on the one hand there are arguments which speak in favour of such a normative power conception, as was the case for the Turkish abolition of the death penalty in 2004 [8], there are important counterarguments as well. Among some of the most telling ones is the normative conditionality in trade and development policies which is viewed by many developing countries as a form of interference in their internal affairs. Aside from that, the EU has also been criticised for not following through when its proclaimed norms such as the protection of human rights or environmental concerns rubbed up against economic interests [62].

It is in this light that this article critically addresses the NPE concept in the case of the EU’s own discourse about environmental migration. As such, environmental migration binds together two types of norms the EU ostensibly stands for, that is, environmental/climate protection and human rights. In fact, the EU has been securitising climate change as a ‘threat multiplier’ ever since 2008 [17,63,64]. Tackling climate change was/is seen by the European Commission (EC) as *this generation’s defining task* [65]. Hence, climate change has been appearing in the EU’s publications connecting the phenomenon to numerous security issues [18]. Phrases such as *new urgency*, *essential* or *the EU cannot do this alone* implying higher stakes were added too [63]. The 2016 EU Global Strategy named climate change among the main current threats *endangering our* [EU] *people and territory* [64]. Moreover, by describing climate change as an existential threat, the EU has attributed itself a role of an active player in the fight against it. It has also acknowledged the existence of environmental migrants as a result of climate change and environmental degradation. In turn, it is meaningful to ask if/how the EU securitises such human beings who are deemed as existentially threatened by the environment.

## The threat-multiplier and the referent object

The type of securitisation I refer to in this analysis and which in this article is connected with the EU’s normative assertion is inspired by the Copenhagen School of Security Studies, particularly the writings of Buzan, Wæver and de Wilde in 1998 who suggested a new and broader understanding of security, that is, from the traditional military and political issue areas, to economic, environmental and societal ones as cited in Vaughan-Williams and Peoples [16], as well as a bigger number of relevant actors involved. Crucial within their approach is the intersubjective nature of security issues [66], which is created by an actor and might be based on objective or subjective arguments. Buzan et al. explained that taking an *issue* and turning it into a *security issue* requires *securitisation* which is a process that is built from multiple identifiable parts. Firstly, a *securitising actor* must make a *securitising move* (via a so-called *speech act*) in which they present an *existential threat* to a *referent object*. Speech acts in this sense are the basis of communication. They are the utterances which lead to *actions* [16] taken by Buzan et al. from the speech act theory. Secondly, the threat must in turn be solved by means of *extraordinary measures*. In addition, a specific *audience* who is the receiver of the speech act, must be *convinced* of the need for the measures to be taken [15]. As Buzan et al. pointed out, this is ‘… the move that takes politics beyond the established rules of the game and frames the issue either as a special kind of politics or as *above politics*’ [15]. Although Buzan et al. explained that the goal in politics is not securitisation, but rather de-securitisation, there are debates among academics about whether, in some situations, securitisation may serve a morally just (normative) cause [67–69]. After all, Wæver himself admitted that there might be scenarios sufficiently worrisome, in which securitisation will be a responsible reaction to block *the worst* [68]. In this sense, Bettini pointed out how securitisation needs apocalyptic narratives. Without them, the securitisation discourses lose much weight [21]. At the same time, Hartmann warned that playing with fear is like playing with fire, because the consequences of securitisation can be hardly controlled [52].

Considering the EU’s international appeals and explicit moral justification for the fight against climate change and the general interest in human rights protection, it is argued here that the EU has taken such a *just cause* (normative) approach of trying to *prevent the worst*, and that the intense justification of the fight against climate change serves as the foundation for the extraordinary measures in the securitisation. On top of that, other actors such as environmentalists [70], human rights activists or academics [9,19,29,38,71] reiterated the EU’s own normative expectations and stressed the need to act on environmental migration. The issue was brought up also by the European Parliament, which requested several studies [7,46,56,57,72]. Nonetheless, what is questioned in this article is to what extent did the EU pursue and fulfil the normative expectations it created for itself and received from others when it addressed the environmental migrants? Following the analysis of the past 20 years of discourse provides a contrasting picture to the normative image.

## Environmental migration and the EU discourse

With the NPE and securitisation theory from the Copenhagen School in mind, the basis of the discourse analysis needs to be set out, a method frequently utilised for research of migration discourses. As indicated by van Dijk, migration discourses do not merely describe migration, they also constitute it as a phenomenon [26]. Depending on how the issue is defined, that is, who is the referent and what is the threat, such discourses may take on strong anti-migrant nature, underline antidemocratic or antihumanitarian rationales and measures [21,52]. Often, ‘problems of the South’ gain attention once they are portrayed as ‘threatening Northern security/interests’ [17,20]. A critical analysis of the normative EU discourse on environment and climate change is thus called for, as it mentions the purported environmental migrants.

Based on the writings by Jones [73], Machin and Mayr [22], van Dijk [26], Wodak and Meyer [25] and van Leeuwen [23,24], this paper assessed numerous EU positions through seven discursive properties, namely the context, structure, style, format/mode, modalities, descriptions and implications, each of them having several analytical subcategories, which may be viewed in Table 1.

**Table 1.** Seven discursive properties derived from literature and analysed in the key documents

Discursive property	Analyzed segments	Sub-categories
Context	International – EU	
Structure	Length of document	
	Number of references	
	Positioning of references	
Style	Author of document	
	Recipient of the document	
	Channel of publication	Personal/Impersonal
	Situation for which a document was published	
	Topic of the document	
	Reference to Actor	Personal/Impersonal
	Form of document	Formal/Semi-formal/Informal
Format/Mode	Argumentation/Description/Exposition//Narration	
Modalities	Nouns/Adverbs/Adjectives/Verbs	
	Meanings: Ability/Certainty/Conditionality/Desirability/Frequency/Future/Necessity/Obligation/Permission/Possibility/Quantity/Willingness	Degree: High/Medium/Low
Descriptions	In reference to the actor, referent object, threat	
	Presence/Absence	
	Positivity/Neutrality/Negativity	
	Agency: Active/Passive	
	Specificity/Generality	
	Gravity: Seriousness/Neutrality/Triviality	
	Complexity/Danger/Norm-response/Neutrality	
Implications	Amplification/Antrophomorphism/Connotation/Emphasis/Euphemism/Hyperbole/Idiom/Juxtaposition/Metaphor/Metonymy/Oxymoron/Modifier/Parenthesis	Sentiment: Positivity/Neutrality/Negativity

The analysis itself was qualitative, with a sample of 20 documents chosen for the 20-year period, based on a combination of random selection and literature review. All data were collected from the official EU websites and databases, but only those stating official EU positions were included, resulting in a well-rounded corpus, see Table 2. Subsequently, the discourse segments which mentioned environmental migration were classified according to the securitisation components identified by Buzan et al. [15]. Therefore, the EU was defined as the securitising actor, climate change as the existential threat and environmental migrants as the referents. The analysis focused on official formal and written published documents (speech acts) as more long-lasting than oral statements. What is more, the audience receiving the speech acts was classified as the broader internal EU population. Increasing investments into research, early-warning systems and resilience/adaptation building mechanisms were categorised as extraordinary measures. Based on these conditions, the analysis produced findings which challenge the NPE image the EU presents.

**Table 2.** A representative data corpus consisting of 20 key EU documents

Year	Title	Type of source
2000	Push and Pull Factors of International Migration	Report
2003	A Secure Europe in a Better World	European Security Strategy
2006	Development and Migration	European Parliament Resolution
2007	Climate Change and International Security	Paper
2008	Climate Change and International Security	Paper
	Report on Implementation of the European Security Strategy: Providing Security in a Changing World	Report
2009	A Secure Europe in a Better World	Implementation Report
	Stockholm Programme: An Open and Secure Europe Serving and Protecting the Citizens	Programme
2011	Council Conclusions on EU Climate Diplomacy	FAC Conclusions
2013	Action Plan for Resilience in Crisis Prone Countries 2013–2020	Commission Staff Working Paper
	Climate Change, Environmental Degradation, and Migration	Commission Staff Working Document/Communication
2014	An Open and Secure Europe: Making It Happen	Communication
2015	A European Agenda on Migration	Communication
	2015 State of the Union	State of the Union
2016	Shared Vision, Common Action: A Stronger Europe	EU Global Strategy
	Towards a Reform of the European Asylum System and Enhancing Legal Avenues to Europe	Communication
2017	White Paper on the Future of Europe	White Paper
	A Strategic Approach to Resilience in the EU’s External Action	Joint Communication
	The New European Consensus on Development ‘Our World, Our Dignity, Our Future’	EU Strategy
2019	The European Green Deal	Communication

## A (non-)normative EU

The primary object of investigation was the context in which the EU has been forming its own discourse about environmental migration. As already implied, the analysis has shown a correlation of the international and European deterministic/alarmist context in the early 2000s. I suggest this correlation because of multiple cases where in 2006 the European Parliament (EP) took notice of the UNHCR predictions [74], and in 2007 the Commission used references to both the UN prognosis and the German Advisory Council which in turn referred to the Stern Review and Myers’ Environmental Exodus [75]. Equally, the 2010s showed a shift toward a rather reserved, pragmatic and less doom-laden approach both globally and in the EU. Among the most significant realisations at the time for the EU was that environmental migrants are most likely to stay in the ‘Global South’. Another finding was that predicting precise numbers of such people is extremely difficult due to a combination of driving forces [27], as also implied by the more current academic discussions [39–46]. Similarly, in 2015 a UN university study admitted that there are no reliable estimates, neither for the present nor for the future, due to the lack of intra-country data and the complex combination of drivers [17,54,76]. In turn, the EU started investing into research on the topic, particularly the creation of methods for how to assess the likelihood of environmental displacements around the world [29]. Likewise, the EU has made numerous funding calls for research projects on climate change and migration as part of the Horizon 2020 program [39]. The Commission’s recognition of the previously ‘faulty’ methodologies, which omitted the multiplicity of drivers, was especially important in this regard [27]. The EC explanation was that ‘… policymakers need to know the magnitude of environmentally induced migration to be convinced of the importance of the phenomenon and to design action’ [27]. What is more, the Union continued funding environmental migration research over the years [77–79] and started referring to assumingly more sophisticated methodological approaches such as the 2011 UK Foresight study, which implied that environmental causes do not have direct effects on migration. Rather, the British study claimed, that they tend to be combined with social, political and economic drivers [27]. Consequently, the EU invested in multiple programmes aimed at adaptation and resilience building in countries of migrants’ origin as well as transit. Other projects dealt with data collection, scenario assessments, and strengthening of cooperation with third countries to prevent flows towards the EU [27,80–83]. Yet, funding of projects has fluctuated over time. Moreover, it came from a multitude of sources, for example, co-funding by EU with member states or other organisations, and a variety of instruments. Clear information about how many projects/studies relevant to environmental migration the EU was/still is involved in was not found. I thus infer that such scattered information about the EU’s involvement makes it difficult to track what the EU did/does. Another identified limitation was a lack of clarity about how much the EU has spent, as not all projects stated the allocated funds. Thus, I could not establish any accurate trend in the expenditures. The third part of the issue was related to one of the requirements of securitisation [15], that is, the audience’s approval for extraordinary measures. Considering the complicated EU structure and decision-making processes, as well as the nature of the assumed audience (EU population), this would require at least a Eurobarometer survey on the topic. Nonetheless, for the 20 years covered, I was not able to find any dedicated questionnaires. Besides, neither the surveys on migration nor climate change included questions about environmental migration. The audience and its approval/disapproval in the topic of environmental migration, I infer, was thus omitted from the securitisation equation.

In addition to the context described above, I analysed the other six discursive properties (structure, style, format/mode, modalities, descriptions, implications) that delved into the contents of the speech acts and the meanings hidden between the lines alike. Starting with the first, the structure initially showed that higher frequencies of references dedicated to environmental migration were rather uncommon in the EU discourse. Only one document from 2013 was fully devoted to the topic. It addressed the complexity of the phenomenon, the threats climate change poses to environmental migrants, and the EU’s actions taken in the past as well as recommendations for the future. It might be noteworthy that 2013 was also the year in which the Warsaw International Mechanism for Loss and Damage associated with Climate Change Impacts was established at the Conference of the Parties (COP) 19, which is a series of UN Climate Change Conferences of the Parties to the UN Framework Convention on Climate Change [84]. Nevertheless, the remainder of the sampled documents contained only short, and vague references to the topic. Those references were scattered throughout the texts under varying headings such as *analysis*, *climate change*, *global challenges*, *resilience* or *united approach of the Union toward climate change*, sometimes as a few out of tens even hundreds of the items covered. Moreover, they were usually located in the main body, which, in analyses of texts, is considered as less attention-catching for the audience compared to information included in titles, introductions or endings [26]. In line with the alarmist–pragmatic contextual changes observed in the context, the period until 2013 has been more dominant in terms of the structural factors than the later years. All (three) documents scoring the highest on the reference scale were found up to 2013, which could resemble a more securitised discourse in the initial stage, which in turn points to a decreasing prioritisation afterwards.

In terms of the second property, that is, style, the analysis showed that throughout the whole period the EU has been predominantly addressing itself in an impersonal manner (*the Union*, *the EU*, *the Member States*, *the Council*). There have been only four documents where more personal indications to the EU and its parts appeared (*we*, *our*, *I*), implying a deeper connection to the issue. Thereupon, I suggest, the EU uses a predominantly formal style in its discourse to appear as an impersonal expert authority without close relations to the audience or the referents. According to existing discourse literature, this may be interpreted as a tool of professional persuasion [26,73,85]. In addition to this, the analysis of the used formats emphasised that the EU used mainly argumentative and expositional formats to convey its messages about the topic. In the earlier period (2000–2014), documents were dominated by exposition, meaning references were fact-based, usually relating to the causes, effects, problems and solutions for climate change on migration. Implications on different states, regions or the EU as a whole were likewise included. Post-2014 the approach changed, however, and argumentation became more frequent. From this point on, the EU’s arguments were stressed through opinions, requests, recommendations, positions, commitments and promises. According to the Copenhagen School, the securitising actor should be persuasive toward the audience to achieve the desired results. What can be thus said is that until 2013 the EU attempted to securitise particularly through the information about environmental migration. Yet later, the discourse became more argumentative, supporting the Union’s responsiveness, and making it more normative. Considering the changing international context at the time, which was firstly deterministic/pessimistic about the phenomenon and its effects, but as time went on became more pragmatic, I therefore suggest a plausible parallel between the EU and international discourses. This could have been the result of the new methodologies and data available; however, more research is necessary to confirm a finite causal connection between the two. A round of interviews with EU experts who were involved in the discourse making in that period would be particularly meaningful for establishing such a link.

By the same token, my assessment of the next discursive property, the modalities, showed further correlation with the changing context. Overall, from the data I derived 12 categories from modality meanings. Listed according to the frequency of appearance these were *possibility*, *future*, *quantity*, *necessity*, *desirability*, *ability*, *frequency* and *obligation*. In terms of the degrees, an overwhelming majority of them belonged to the high degree (e.g., *always*, *most*, *have to*), followed by the medium (e.g., *likely*, *some*, *should*) and low degrees (e.g., *possible*, *could*, *may*). Throughout the 20-year period I observed that the lower specificity/frequency/quantity appeared mainly between 2000 and 2013, and the medium to higher modalities were spread more evenly across the whole 20-year time span. Consequently, this dispersion made the post-2013 period visibly dominated by the high and medium degrees modal words. I interpret this as a growing certainty and specificity on behalf of the EU within its references, as it was letting go of less certain language over time. Moreover, I argue that the most frequent and obvious reappearance of the two categories *quantity* (2007–2016), was matched by words with a medium-to-high degree such as *much*, *many*, *most*, *some* and *possibility* (2000–2016) resembled by words with a low-to-medium degree such as *likely*, *can*, *could*, *may* or *potential*, which could have been derived from the impact of the earlier alarmist, yet unreliable, context in which the discourse was created. Hence, I connect these modalities with the more securitised period. On the contrary, post-2016 I found the category *future* as the most prominent, mainly relating to what the EU *will do*, followed by the second most recurring category *necessity*, which stated what the EU *has to do*. I thus argue that the overall pattern of the categories can be interpreted as a move to a more normative and task-oriented EU discourse in the later years (post-2013) in relation to the perceived environmental migration phenomenon.

Regarding the EU’s normative self-identification, the analysis has shown corresponding patterns also in terms of inclusion–exclusion and agency activation–passivation [24]. At the centre of focus in the discourse were environmental migrants (referents), albeit they were described as passive and always being influenced by the consistently active threat, climate change. The threat received equal amounts of attention in the speech acts. On the other side, the most absent was the EU (actor), who was more often than not portrayed as an active respondent in the situation. Such positioning of the active actor in relation to the passive referents endangered by the ever-present threat coincides with the ideas about a just securitisation and the normative image of self as a saviour and expert. Furthermore, extraordinary measures were implied through appropriation of policies, need for research, resilience and adaptation building. In general, the references were prevalently specific, which in turn made them more convincing in line with the EU’s image of an expert authority as was observed in the used style before. At the same time, the small frequency of the EU appearance as the actor may imply a form of distancing and limit the involvement of the EU in what happens to the environmental migrants.

Moving on to another discursive property, that is, descriptions which enable uncovering held attitudes [23,26] of the EU about the phenomenon, the analysis showed four different types of narratives. Importantly, I noted a trend whereby negative connotations were found mainly in the earlier data pre-2015. The neutral and positive ones came more so in the later stages. Overall, this corresponds to the 1999–2013 (securitisation) and 2013–2019 (normativity) division with only a slight 2-year delay. On top of that, descriptions of how seriously the situation was portrayed implied a similar pattern. Earlier years (2000–2011) involved descriptions of high urgency, for example, *threaten stability*, *significant impact*, *large-scale*, *intensify*, *aggravated*, *exacerbating* or *very important*. Contrary to this, since 2013 neutrality was more dominant, mentioning both negative impact and positive opportunities of the phenomenon. Additionally, in the discursive property *descriptions*, the category *danger* was prevalent until 2011, but was replaced after 2013 with categories *norm/response* and *complexity*, which often appeared together. This suggests the complexity of the issue, namely that the multiplicity of migratory drivers was appreciated more in the EU as a result of newer research methodologies.

While six of the properties have fallen in line, the seventh property (implications) did not follow suit. Overall, the distribution of rhetorical devices in the EU discourse about environmental migration did not resemble a coherent pattern from which I could draw a clear connection to the results of other parts of my analysis, and thus draw onto the securitised pre-2013 and normative post-2013 periods. What can be said is that the metaphors persistently implied negative images, for example, *root cause(s)*, *driver(s)*, *flow(s)*, *pressure(s)* or *trigger(s/-ed)*, that are typically found also in other migration discourses held by different actors [86]. While two documents (i.e., Towards a Reform of the European Asylum System and Enhancing Legal Avenues to Europe, and The European Green Deal) categorised as positive did appear in the post-2013 period (due to the positive narratives in the devices they incorporated, e.g., the metaphors, euphemisms, metonymies, etc.), the negative implications dominated most of the documents throughout the 20 years and therefore did not underline the securitisation–normativity division observed in the other parts of the analysis. Rather, they resembled the general trend in discourses about migration.

## Failed securitisation?

The overall assessment of this analysis is that in the case of environmental migrants, the EU’s discourse did not meet its own normative goals which are meant for the protection of human rights as well as protection of the environment. At the same time, I argue that the successful securitisation of climate change towards environmental migrants has not reached the required extent throughout the whole 20-year period as it was defined by Buzan et al. [15]. Thus, it can be said that the EU discourse contained merely securitisation attempts. All things considered, the EU attempted securitising the issue mainly during the earlier period, predominantly in a negative/pressing manner. Here I infer plausible influences of international alarmism, although more research is necessary to prove this suggestion. For example, a noticeable characteristic of the references was their lack of consistency and frequency, which was rather surprising considering the EU’s strong focus on climate change over the past decades. Aside from environmental migrants, which appeared to be less important in the discourse, attention was given to other referents of climate change threats, for example, the impacts on the EU territory at large, EU’s citizens, the environment in general, as well as developing countries and the people living there, resource shortages, energy supplies, food insecurity and so on [64,65,87]. For a successful securitisation move, the referent (for this analysis it was the environmental migrants) should be clearly and coherently portrayed to the audience. This was not the case in the EU discourse, however, where the migrants appeared, but were tied with terminology that was rather distant and ambiguous. Neither should the main actor (EU) be as absent as it was in the data, when compared to the referent migrants and the threat. Furthermore, the references to environmental migration were also more often than not brief, and located in the main body of the documents, which made them blend in with the rest of the information. With respect to the extraordinary measures that are crucial to any securitisation, the EU pursued mainly extensive research into the topic, and invested into resilience building and early warning systems via already existing tools, for example, foreign and development policies. Yet, no new tools were found, and it was not possible to find exact expenditures on these activities either. Arguably, if an outstandingly large amount has been spent on such extraordinary measures, this would most likely be publicised in order to underline the importance of the cause as is the case in all other crises. Again, this was not the case, hence it is more reasonable to speak only of securitisation attempts. What is more, likewise, the absence of a feedback instrument such as Eurobarometer surveys on the topic undermines the role of audiences in securitisation. Besides, there were only two consistent discursive properties (negative implications and formal style) observed through the whole 20-year period, which in itself shows a lack of a finite and coherent EU position toward the phenomenon. This gets in the way of conveying a convincing securitised message to any audience, which also builds on the Copenhagen School’s own observations that securitisation of climate change has supposedly not reached exceptionality just yet [17].

Notably, post-2013 the topic has been declining in importance, evident from its decreasing appearances in the data, the language has become more neutral to positive, and the general approach has evolved to pragmatism as could also be observed in the international context. The changed EU position towards the complex nature of factors which cause environmental migration can be thus viewed as parallel to contemporary scientific findings. Bettini [21] argues that this can have positive de-politicising effects because much of the early international discourse was not based on facts. Instead, mere hypotheses of catastrophic outcomes used to be presented as given [21,52]. On the contrary, this position is being questioned. Klepp points out that such a shift undermines the importance of the root environmental causes, and instead shifts attention to other causes, for example, social or political [20]. Simultaneously, Boas et al. [39] note the importance of the multifaceted motives for human mobility (and immobility), and the fact that climate change reinforces other existing drivers, rather than being the only one responsible. Mobility is thus seen as multicausal. Once again, this supports the claim that the change of methodologies and international context likely impacted EU discourse. Nonetheless, in order to definitely confirm such causality, further research is crucial.

With regard to the ideas of a just or normative securitisation, it remains the case that the EU pursued securitisation attempts of climate change and its impacts in a normative manner. It has proclaimed its dedication to continually do so and to convince partners beyond the EU to follow suit. Even so, the normative securitised fight against climate change did not reflect onto environmental migrants as much as could also be expected from the human rights perspective. In the references, the normative commitment to help or protect them has appeared only since 2014, that is, in the period with lesser focus. This might have been due to a number of factors such as either the uncertainty about how environmental migration might develop and how it should be dealt with, or how such migration could be prevented through resilience/adaptation measures. Simultaneously, the commitment mentioned was not always binding (*we/the EU will*). Instead, it often relied on modal verbs of lower degrees like *should*, *ought*, *need to*, *must*, *shall*, etc. While the use of modal verbs implied what is, for example, the right/moral/normative thing to do, what is possible or desirable, they have not consistently done so, at least not in the earlier period (pre-2013). Only since 2015 has the EU turned more to expressions about what it will actually do, rather than what it should/might do. Nevertheless, the norms (what should/will be done by the EU for environmental migrants) have been limited, which is a repeated finding across research done on the topic. For example, the EU has not included any promises that would involve advocation for the rights of environmental migrants or a creation of a separate protection status [20], whether in EU legislation or in international fora such as the UN. As implied earlier, European countries and the EU alike did not seek answers to the conceptualisation question, nor any additional legislative changes [41,42]. Although this has been clearly voiced as a desired step by the states most affected by climate change in the Global South, and it was even mentioned in an EC publication in 2007, as well as by the EP in 2006. Equally as meaningful is the fact that where environmental migrants were referenced, the possibility of permanent damage to them or their homeland has not been acknowledged, a problem that might be particular to inhabitants of Small Island States in the South of the Pacific Ocean [49,88,89]. Here, there is an additional debate addressing the right to cultural heritage of people inhabiting Small Island States that are being overflown by rising ocean levels. Some question whether the islanders should not be provided with territory from other states, possibly those who contributed the most to the climate change [90]. Such proposals have not been mentioned in the EU discourse.

## Post-2019

While the analysis has covered the initial 20 years of European Discourse up until the creation of the von der Leyen Commission and publication of its European Green Deal, there have been additional developments in the past three years which ought to be addressed. Most notably, since 2020 the world has been significantly impacted by several waves of the coronavirus (Covid-19) pandemic. While the EC president claimed that the Green Deal remains the pinnacle of pro-environment policy and should remain on top of the EU agenda despite the pandemic, this document did not give much attention to environmental migration [91]. The 2020 EU New Pact on Migration and Asylum, a centrepiece for contemporary migration policies in the Union, did not include any direct references to climate or environmentally induced migration either [92]. What is more, during the past few years the world has experienced hundreds of devastating environmental disasters [55].

Despite the pandemic, the discourse around environmentally driven migration continues on the international as well as European scenes. The World Bank and UN agencies such as the International Organisation for Migration and the UN High Commissioner for Human Rights have been continuously acknowledging the possibility of climate-induced displacements [93]. Similarly, EU research services, especially for the EP, have produced documents addressing the topic in this period, albeit they did not represent the official view of the EU as an institution [55,57,94–96]. In terms of EU involvement, the papers implied the EU should have a crucial role, for it could prevent conflicts and migration waves (to its own shores/borders). After all, it is likely to experience large influxes of *climate migrants*. Thus, the EU should continue with emission mitigation policies, support resilience measures in vulnerable countries and get involved in joint environmental research and knowledge/technology sharing [95]. Some of the papers prepared for the EU, which did not state its official positions, however, acknowledged the importance of a dedicated legal framework for environmental migrants but noted that the EU did not recognise climate stress as reason for seeking a protection status so far [95].

The topic was also recently included in the UN COP conferences. In Glasgow at the COP 26 in 2021, the EU co-sponsored a side event with OHCHR addressing climate impacts as drivers of migration [47]. On the following COP 27 in Sharm El-Sheikh, the EU co-sponsored another event titled *Addressing displacement and migration related to the adverse impacts of climate change: partnerships and integrated approaches for action and support* [97,98]. In regard to this latest COP, the EP produced a resolution in October 2022 which suggested that ‘*…* global action to reduce GHG emissions could dramatically slow the rise in internal climate migrants by as much as 80% by 2050’ [99]. While the resolution acknowledged negative impacts on human rights of the affected persons, no further specificities were included [99]. At the same time, the conference was a turning point in the sense that the most affected developing countries unitedly demanded funding from the biggest emission producers to prepare for and recover from climate-driven disasters. Likewise, people who were displaced due to environmental/climate factors came and spoke out at the conference. They also demanded to be included in negotiations about their fate and to have seats at the next COP 28 table [100].

## Conclusions

In conclusion, the results of this analysis have implied that while the EU still pursues international protection of human rights and securitises climate change as a just cause, the so-called environmental migrants do not appear to be a primary concern in the related discourse. Instead, a multiplicity of other threatened subjects is at the forefront. While environmental migrants are still being mentioned from time to time, and there have been observed proclamations by the EU to act on behalf of such groups of people, they were rather occasional, typically nonbinding and general in nature. This analysis thus showed that successful securitisation as described by the Copenhagen School was not achieved in this discourse. What is more, the times when the securitisation attempts were more prominent (1999–2013), the narratives related to environmental migration were rather negative. They became normative only once new data suggested that environmental migration might not have as significant and negative impacts on the EU as was initially believed. The reason behind it could be the change in international discourse from alarmism to more pragmatism caused by new qualitative methodologies that addressed the complexity of factors behind the phenomenon, and consequently produced different data. Given the multitude of studies prepared for the EU institutions, as well as the EU’s investments into further research in affected areas, an evidence vacuum which might have given reason for initial (unfounded) fears is now less likely. I argue that the initial predictions about environmental migrants’ numbers in the coming decades were no longer certain, and thus seemingly decreased the urgency of the issue in the Global North. Also, at the point when normativity appeared in the EU discourse (post-2013), the topic has been receding into the background. Still, in the instances when environmental migration was included, the EU has addressed it from its own standpoint, and did not respond to such requests made by the referents such as a new legal protection status. Neither has the EU bound itself to concrete actions. More so, it continuously focuses on funding research, prevention, resilience and adaptation measures dealing with climate change impacts while keeping affected people in place. I therefore argue that the EU normativity, as it has been described by Manners [8,9] and requested by Green MEPs along with civil society from the EU in the early 2000s is difficult to claim, despite the EU’s growing interest in humanitarian and environmental protection. According to the findings, the self-set normative standards in the nexus of the two issue areas are not being met when it comes to environmental migrants considered as referents of climate change. All in all, there is still a long way to go in international relations when it comes to the analysis of migration and the environmental drivers behind it. The topic will certainly remain current due to continuous climate change and environmental degradation. It is questionable though whether the EU will retain the notion of environmental migration as a separate type of human movement, and there remains considerable uncertainty as to which actions will be taken in the future. A significant obstruction may be the difficulty in establishing direct causal links between the environmental drivers and the human movement, as well as a clear means of identifying such migrants.

## Data Availability

The datasets generated during and/or analysed during the current study are available from the corresponding author on reasonable request.
